# A Case of Twiddler’s Syndrome: A Rare Complication of Pacemakers

**DOI:** 10.7759/cureus.71923

**Published:** 2024-10-20

**Authors:** Jhon Navarro-Gonzalez, Marcelo Durán Caamaño, Cristóbal Lara Pedreros, Karina Sepulveda

**Affiliations:** 1 Emergency Department, Hospital Las Higueras, Talcahuano, CHL

**Keywords:** pacemaker complication, pacemaker lead displacement, pacemaker lead migration, single-chamber pacemaker, twiddler's syndrome

## Abstract

Twiddler's syndrome is a rare complication of cardiac pacemakers, which involves the retraction of the lead with rotation inside the implant pocket. This article presents the case of a 54-year-old female patient, who was admitted to the emergency department for chest pain and involuntary contractions of the left pectoral and left upper extremity. The patient is evaluated by a cardiologist specializing in electrophysiology, confirming the Twiddler's syndrome diagnosis.

## Introduction

Twiddler's syndrome is a rare but potentially dangerous late complication of definitive transvenous pacemaker placement, which was first described in 1968 [1]. It is characterized by the retraction of the electrode lead, often secondary to the patient's external manipulation of the device, which causes the leads to migrate and wind up in the insertion pocket of the rest of the circuit [2,3,4]. This phenomenon can cause a dislocation of the electrodes, resulting in inadequate stimulation or a complete loss of function of the pacemaker [5,6].

In addition to external manipulation, several risk factors are associated with Twiddler's syndrome. Obesity is a significant risk factor, as excess adipose tissue can allow for greater movement of the device inside its subcutaneous pocket. Due to tissue laxity, connective tissue conditions, such as Ehlers-Danlos syndrome, can also predispose patients to this complication [7]. Other factors include inadequate surgical technique during initial implantation and an excessively large subcutaneous pocket for the pacemaker generator [8].

Early recognition and proper management, such as repositioning the device, are crucial to prevent further complications and ensure the device continues to function correctly [9].

## Case presentation

A 54-year-old female patient was admitted to the emergency department due to a clinical condition, which began approximately 24 hours prior, characterized by constant chest pain at rest, nonanginal, of electrical nature, radiated to the left shoulder area, associated with rhythmic involuntary movements of the left pectoral and left upper extremity. During interrogation, the patient did not report nausea, vomiting, or neurovegetative symptoms that suggest acute coronary syndrome. She denied manual manipulation of her pacemaker.

Her medical history includes a history of non-ischemic dilated cardiomyopathy and permanent atrial fibrillation, carrier of a mechanical valve prosthesis, hypothyroidism, and the installation of a Medtronic Sphera spsr01 single-chamber pacemaker (VVI mode) two months ago with AV node ablation in the context of sick sinus syndrome and atrial fibrillation.

During the physical examination, the patient was alert and oriented, although visibly in pain and suffering involuntary contractions of the left pectoral muscle, however, without loss of muscle strength or other abnormal findings during neurological examination. A blood pressure of 114/79 mmHg and a heart rate of 96 beats per minute were registered, and the patient was hemodynamically stable and well perfused with normal capillary refill time. During cardiac auscultation, no murmurs were heard, regular rhythm in two tones. Pulmonary examination showed no pathological findings, symmetrical respiratory sounds, and oxygen saturation of 96% with environmental FIO_2_ with preserved ventilatory mechanics. She was afebrile and the rest of the physical examination showed no pathological findings.

When the patient was connected to the cardiac monitor, pacemaker spikes were observed, which did not correlate with the myocardial depolarization activity (QRS complex).

**Figure 1:**
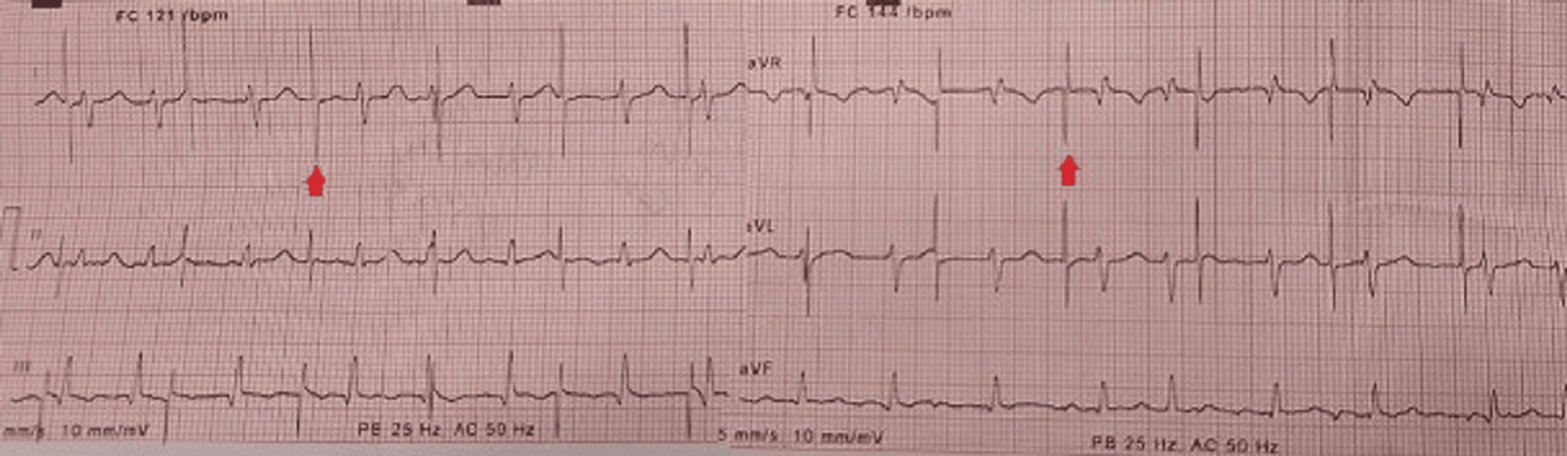
Electrocardiogram without appropriate ventricular capture

**Figure 2:**
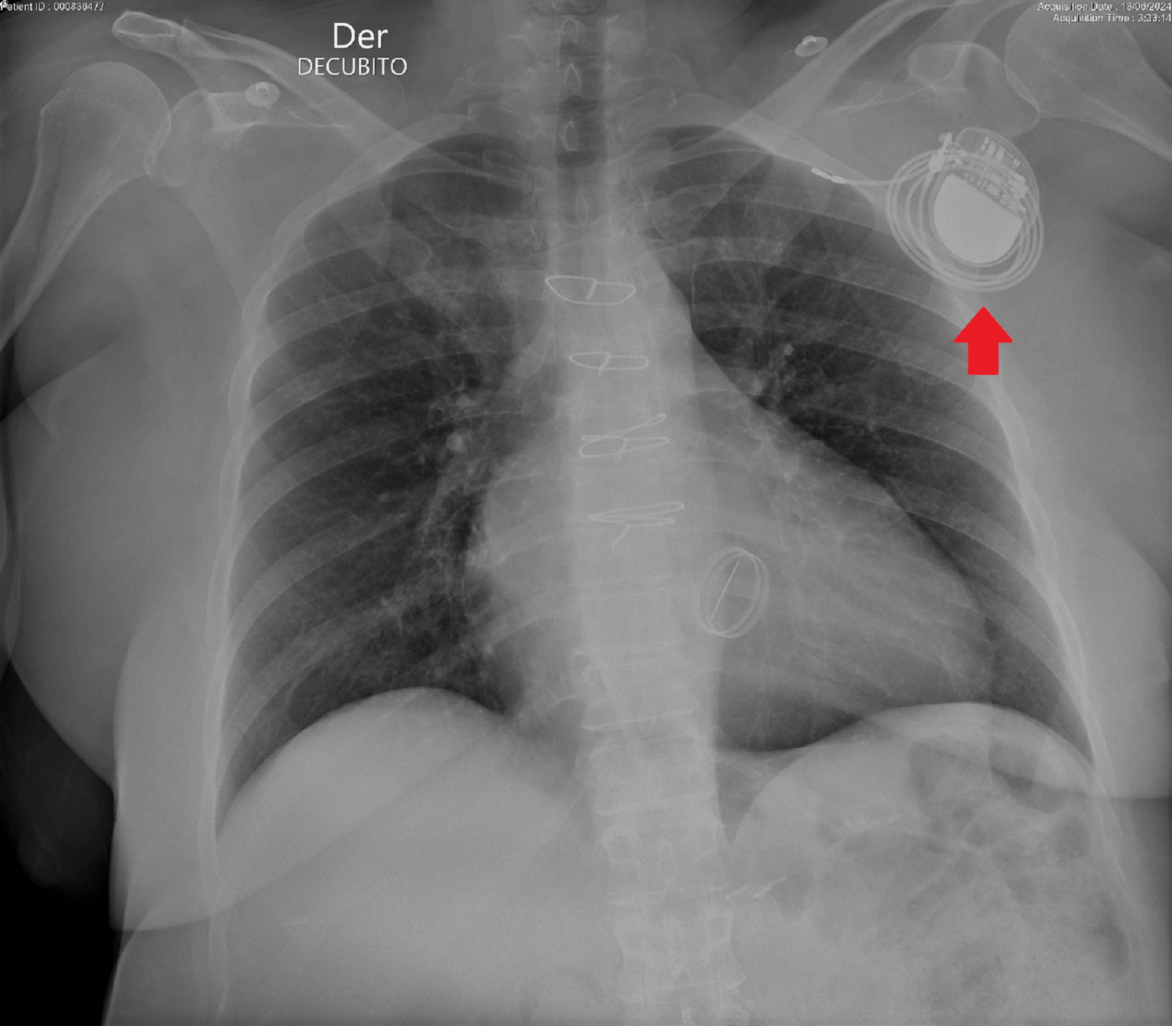
Chest X-ray showing ventricular electrode displacement and twisted lead near the generator

**Figure 3:**
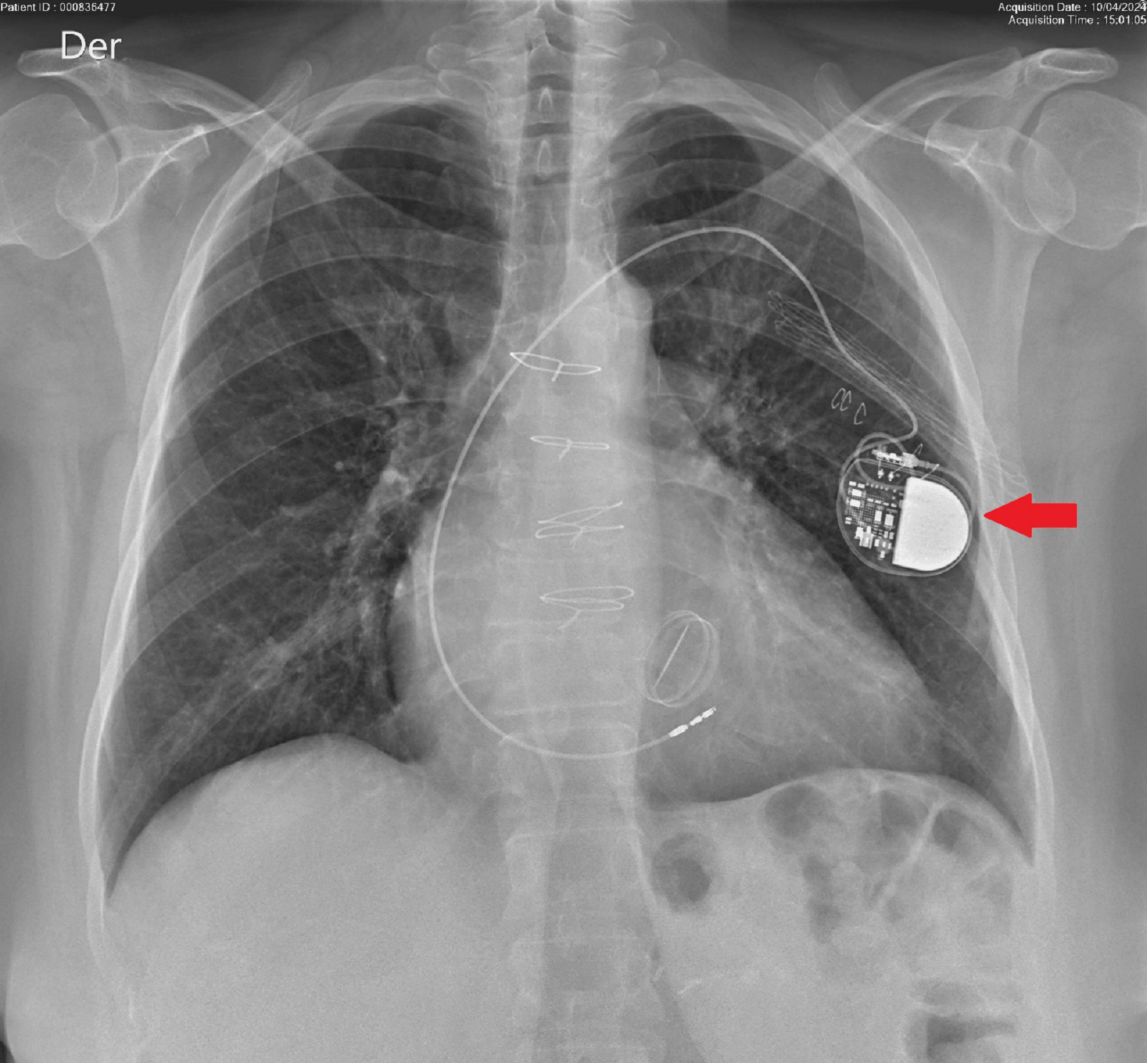
Chest X-ray after pacemaker and ventricular lead repositioning

The electrocardiogram (ECG) revealed a narrow irregular rhythm compatible with atrial fibrillation and pacemaker spikes without associated QRS complexes, showing a ventricular capture failure (Figure 1). A chest X-ray is performed, which shows the ventricular electrode displacement wrapped around the generator, which is indicative of Twiddler's syndrome (Figures 2-3).

As for the laboratory test results, no relevant alterations were found.

**Figure 4:**
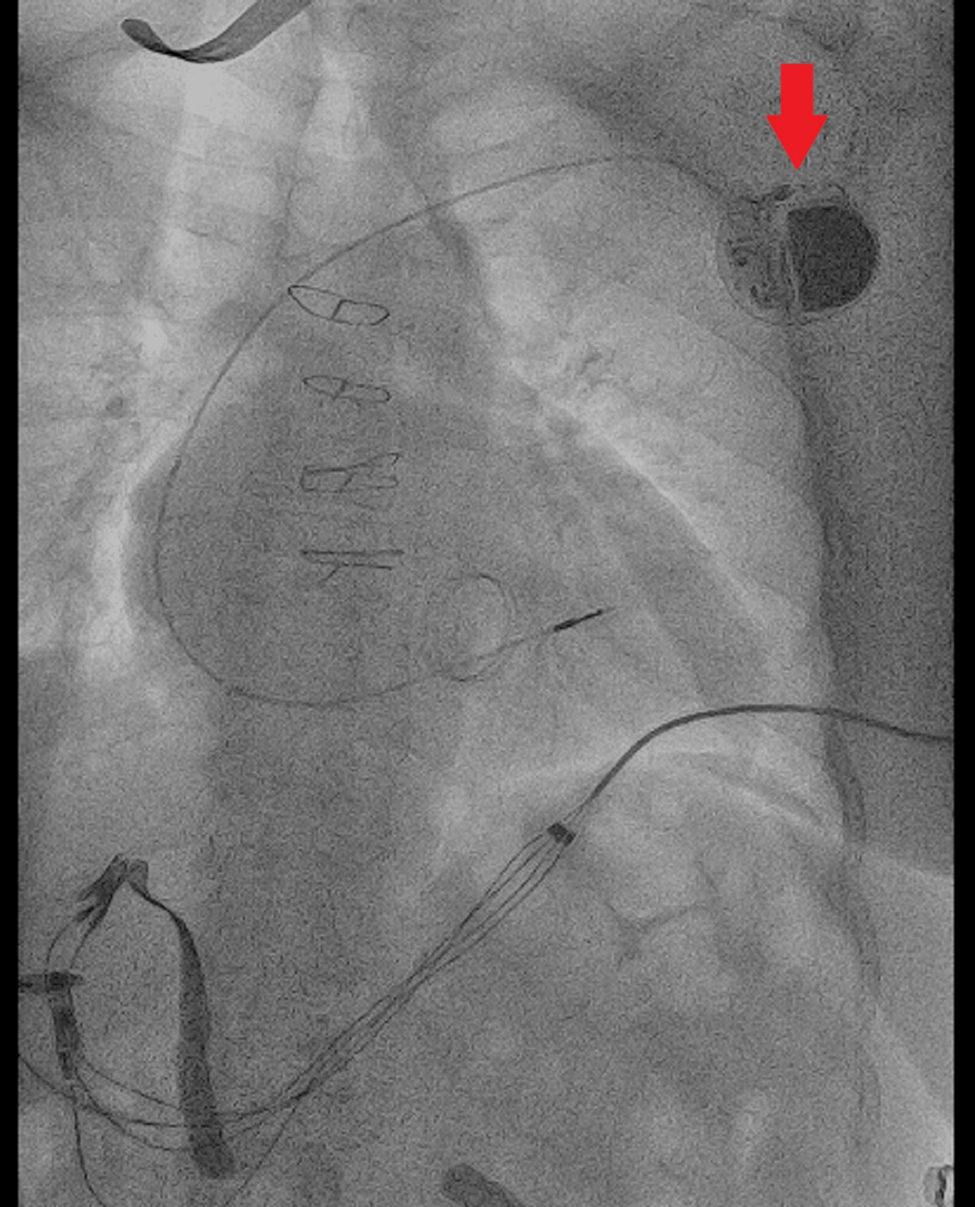
Fluoroscopic image displaying the pacemaker lead with visible coiling and displacement, consistent with Twiddler's syndrome

**Figure 5:**
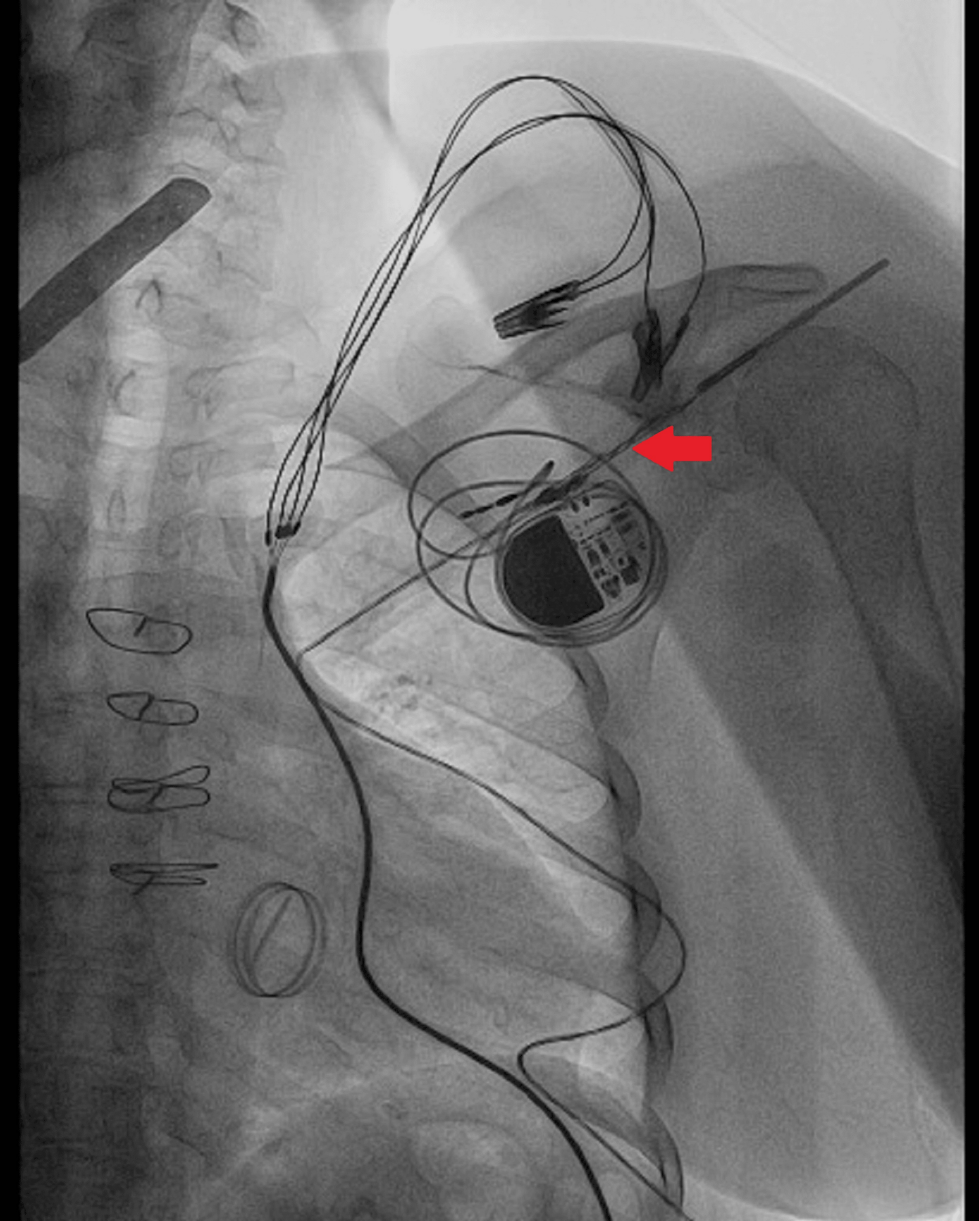
Fluoroscopic view showing the repositioning of the ventricular lead within the right ventricle after the initial displacement

**Figure 6:**
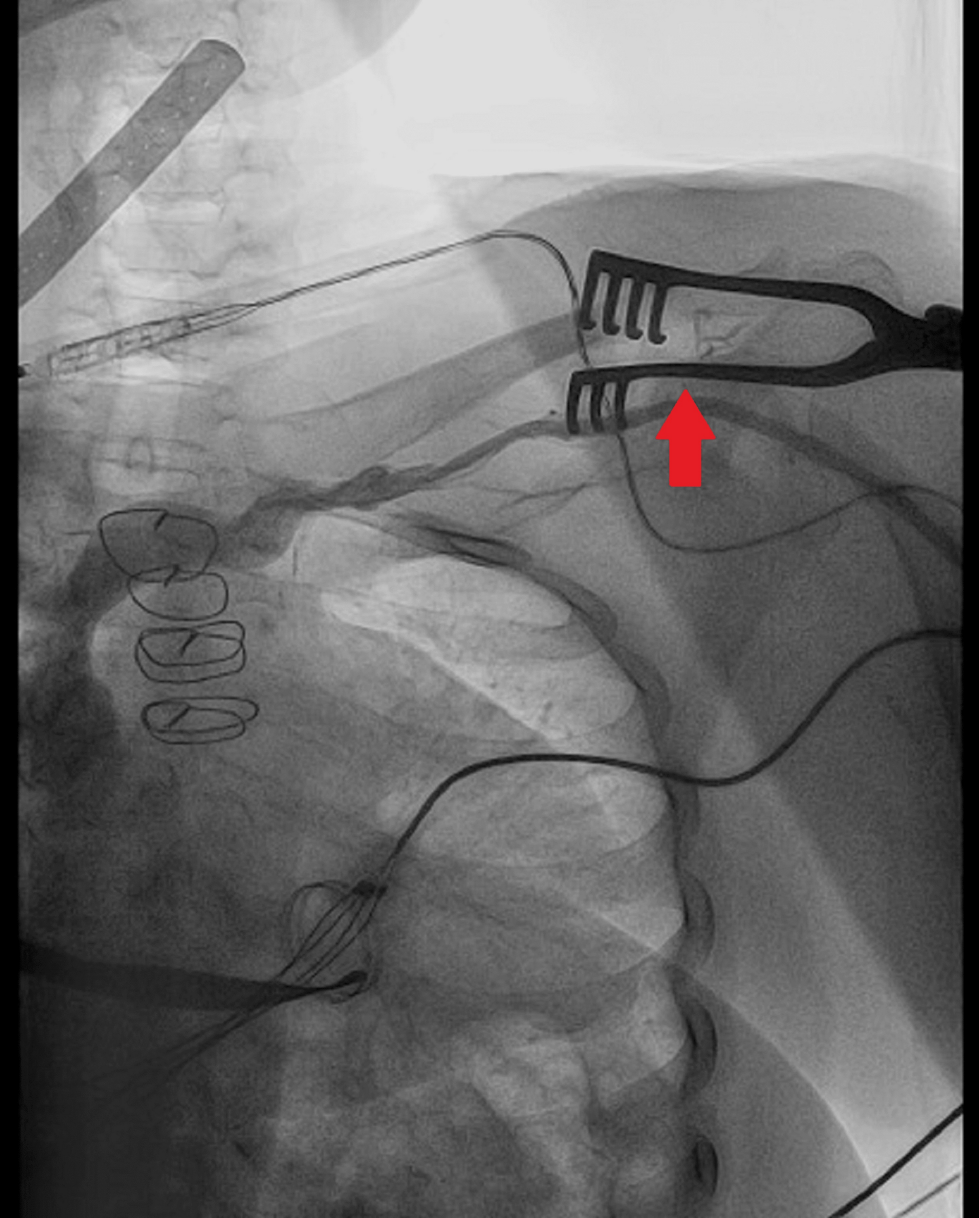
Final fluoroscopic image illustrating the secure fixation of the pacemaker lead, ensuring proper placement post-repositioning

Analgesia was administered using an infusion of fentanyl in low doses, obtaining a good response. The patient was hospitalized for evaluation by the Electrophysiology Department. After a few hours, she was evaluated by an electro-physiologist who confirmed the diagnosis of Twiddler's syndrome and proceeded to deactivate the pacemaker and switch it to OVO mode. Finally, the patient was taken to the catheterization laboratory, where the re-implantation of the device, and suturing of the generator to deep planes are performed (Figures 4-6).

## Discussion

This case describes Twiddler's syndrome in a patient with no history of voluntary manipulation of the pacemaker generator [10]. This is an uncommon complication that occurs after pacemaker implantation, mainly caused by voluntary or involuntary manipulation of the generator [11].

Lead migration can lead to a variety of device malfunctions, such as failure to pace, loss of capture, changes in sensing parameters, increased battery consumption, manifesting as symptoms related to the underlying heart disease for which the device was implanted for treatment, such as palpitations, shortness of breath, or chest pain [12,13]. Although it was initially described in pacemakers, cases have also been reported for resynchronization devices and defibrillators [14], where the malfunction could expose the patient to malignant ventricular arrhythmias and sudden death [2,6,15,16].

The malposition of the electrode can cause ipsilateral phrenic nerve stimulation, resulting in diaphragm activation and abdominal movements. In addition, brachial plexus stimulation can manifest as rhythmic arm movements, as what occurred in this case.

The diagnosis of Twiddler's syndrome is based on a combination of medical history, physical examination, and imaging studies. Anamnesis may reveal a manipulation of the device by the patient, and the physical examination may show abnormal mobility of the pacemaker inside the subcutaneous pocket [17]. Chest X-ray is essential to confirm the diagnosis, as it typically shows the displacement and winding of the electrodes around the pacemaker generator [17,18]. In some cases, it may be useful to perform a fluoroscopy to assess the mobility of the device in real time [19].

The treatment for Twiddler's syndrome is surgical and consists of the removal of the affected device and the reinstallation of the pacemaker [15], as was performed in this case. During surgery, it is crucial to firmly secure the pacemaker generator into its new subcutaneous pocket to prevent recurrences. This may involve creating a smaller pocket and using sutures to anchor the device. Patient education on the importance of avoiding manipulation of the device is essential to prevent future episodes [6]. In addition, long-term follow-up should be considered to evaluate the position of the pacemaker and the functionality of the device.

## Conclusions

Twiddler's syndrome is a complication of cardiac pacemakers that should be suspected in patients carrying these devices, who present involuntary contractions of the pectoral and shoulder muscles, associated with the absence of ventricular capture of the pacemaker to the cardiac monitor and the ECG. Clinical suspicion was very fundamental in this case for a timely diagnosis, which could be confirmed relatively quickly with a chest X-ray and for an adequate treatment, without causing major consequences for the patient.
